# Microecological insight into the microbial structure, key cellulolytic community, and microbial interaction during cellulose degradation in high-diversity and low-diversity communities

**DOI:** 10.1128/aem.02376-25

**Published:** 2026-02-18

**Authors:** Xinyue Zhang, Anna Xian, Xi Zhang, Die Yang, Jingya Zhang, Guangxin Chen, Zhiyong Xing, Jingxue Kang, Kaice Lang, Jiaxin Bao, Hongtao Li, Bo Ma

**Affiliations:** 1 College of Resources and Environmental Sciences, Northeast Agricultural University 12430, Harbin, China; 2 School of Animal Medicine, Northeast Agricultural University 12430, Harbin, China; 3 Guangxi Technology Innovation Cooperation Base of Prevention and Control Pathogenic Microbes with Drug Resistance, Youjiang Medical University for Nationalities 74654 , Baise, China; 4 School of Laboratory Medicine, Youjiang Medical University for Nationalities 74654 , Baise, Guangxi, China; 5 Guangxi Zhuang Autonomous Region Engineering Research Center of Clinical Prevention and Control Technology and Leading Drug for Microorganisms with Drug Resistance in Border Ethnic Areasn, Baise, China; 6 Key Laboratory of the Prevention and Treatment of Drug Resistant Microbial Infecting, Education Department of Guangxi Zhuang Autonomous Region, Youjiang Medical University for Nationalities 74654 , Baise, China; Norwegian University of Life Sciences, Ås, Norway

**Keywords:** dilution-to-extinction, high/low-diversity, cellulose degradation, functional microbial community, key microbes

## Abstract

Inadequate management of lignocellulosic waste poses a risk of substantial environmental pollution. Enriched microbial communities selected from environmental samples can effectively contribute to lignocellulose degradation. Utilizing a lower diversity but equally effective microbial community can enhance the control and efficiency of industrial operations. However, the mechanisms of cellulose degradation and functional microbial interactions within microbial communities with reduced diversity remain unclear. In this study, high-diversity and low-diversity lignocellulose-degrading communities were constructed using the dilution-to-stimulation and dilution-to-extinction methods. The enzymatic activity, community composition, degradation pathways, key functional microbes, and microbial co-occurrence network during cellulose degradation were analyzed in both high-diversity and low-diversity communities at the DNA and RNA level. Results showed that the low-diversity community exhibited a higher substrate degradation rate than the higher-diversity community. The activity of FPase and CMCase in the low-diversity community was significantly higher. *Sphingobacterium*, *Pseudoxanthomonas*, and *Devosia* were key players in the high-diversity community. *Cellulomonas* played a significant role in the low-diversity community. Reducing community diversity strengthens the cooperation among functional microbes. This study can guide the design of functional microbial synthetic communities and also can help to expand the ecological understanding of lignocellulosic waste degradation in synthetic microbial systems.

**IMPORTANCE:**

Microbial community diversity is pivotal in the degradation of lignocellulose. Nonetheless, reducing microbial diversity does not invariably result in decreased degradation efficiency. The utilization of low-diversity communities offers several advantages in industrial applications. Previous studies on lignocellulose-degrading functional microbial communities with low diversity have predominantly concentrated on community composition, with limited investigation into functionality and microbial interaction mechanisms. In this study, we constructed microbial communities with high and low diversity to investigate their efficiency in lignocellulose degradation and to elucidate the microbial ecological mechanisms. Our findings indicate that communities with low diversity decreased microbial competition and altered the composition of key functional microbes during the lignocellulose degradation process, thereby enhancing the efficiency of lignocellulose degradation. Investigating the microbial ecological mechanisms underlying lignocellulose degradation in both high- and low-diversity microbial communities can aid in the design of synthetic functional microbial communities and significantly contribute to the bioconversion of lignocellulosic waste.

## INTRODUCTION

The amount of crop straw produced in the world is huge every year. The inadequate disposal of straw resources will inevitably put enormous pressure on the environment (1). The efficient utilization of lignocellulosic waste materials (including rice and maize straw) is essential for sustainable development (2). The adoption of microbial techniques for lignocellulose degradation has recently gained considerable attention due to its low cost, environmental friendliness, and operational simplicity (3). However, there is a growing recognition that the complex process of lignocellulose decomposition, which necessitates the combined action of multiple enzymes and pathways, is rarely concentrated in a single strain (4). Consequently, the utilization of microbial communities is regarded as an effective approach to facilitate lignocellulose degradation, exhibiting superior degradation capabilities in comparison to monoculture methodologies.

The design and/or selection of microbial community with a targeted function can be achieved through two approaches (4). The first approach, known as the “down-top strategy,” involves designing the community by combining several known microbes to achieve the desired functions. For instance, Bohra et al. (5) built a lignocellulose microbial community that can synergistically interact to enhance cellulase production. Prigigallo et al. (6) artificially constructed microbial consortia for biological control purposes, while Ge et al. (7) designed synthetic microbial communities specifically for remediation of soils contaminated with heavy metals. The cooperation between microbial communities obtained in this way is limited. The second approach, known as the “top-down design,” entails selecting microbial communities from natural environments and cultivating them in a specialized culture medium that promotes the survival of the fittest through sequential transfers (8). The community obtained in this way exhibits a greater number of members, more complex interrelationships with each other, and enhanced robustness (9), making it highly applicable in diverse settings.

Consequently, in the last decades, numerous lignocellulose-degrading microbial consortia have been identified and functionally characterized after enrichment from environmental samples (10–13). Although subjected to stringent environmental selection, these microbial communities retain substantial phylogenetic diversity. This is attributed to the functional redundancy in natural microbial communities, wherein multiple taxa can perform similar metabolic roles (14). In contrast to high-diversity (HD) microbial communities, low-diversity (LD) microbial communities offer numerous advantages. These include enhanced predictability, reduced resource and cost requirements, and optimization of specific metabolic pathways due to decreased competition among species. Thus, the adoption of microbial community with reduced complexity, but equivalent efficiency, has the potential to enhance the control and efficiency of industrial operations.

For instance, a minimal microbial community capable of methane production from lignocellulose has been designed and characterized (15). Puentes-Téllez et al. (2) designed an efficient microbial community specifically for the degradation of sugarcane bagasse. Similarly, Díaz-García et al. (16) selected a minimal and versatile bacterial community with lignocellulolytic capabilities. However, these studies primarily focus on assessing lignocellulose degradation efficiency and characterizing microbial community structure but fail to elucidate the complex interactions between microbial communities of varying diversities during the degradation process. A comprehensive understanding of these interactions is essential to identifying key species that drive the degradation process and informing the development of optimized cellulose-degrading microbial communities (17).

In this study, an HD lignocellulose-degrading community was enriched using the dilution-to-stimulation method, and an LD community with increased degradation efficiency but reduced diversity was identified through dilution-to-extinction. The activity of key enzymes related to cellulose degradation was assessed in both HD and LD communities. Meanwhile, the changes in functional microbial community composition and metabolic functions associated with cellulose degradation were revealed. In addition, the interactions among functional microbes in communities with high and low diversity were investigated, and the key functional microbes were identified through a combined metagenomic and metatranscriptomic analysis of the degradation process. The results of this research have the potential to inform strategies for the sustainable utilization of cellulose resources and facilitate the high-value utilization of cellulosic biomass in the future.

## MATERIALS AND METHODS

### Substrate and medium

Agricultural residues (rice straw and maize straw) were collected from Teaching and Research Farm of the Northeast Agricultural University (Harbin, China). The agricultural residues were shade-dried for 2 weeks before use. The dried materials were milled and sieved through a 1 mm mesh. To remove soluble oligosaccharides while retaining the complete lignocellulose (including lignin, cellulose, and hemicellulose), the pulverized materials were thoroughly washed with distilled water (once), 70% ethanol (twice), and distilled water (twice). Materials were oven-dried at 50°C until a constant weight was achieved and subsequently stored at room temperature until use. Lignocellulose-containing bacterial medium (LCBM) was prepared with 1% lignocellulosic substrate (containing 0.5% maize straw and 0.5% rice straw) with mineral salt medium (MSM) (2 g/L K_2_HPO_4_, 7 g/L Na_2_HPO_4_, 1 g/L (NH_4_)_2_SO_4_, 0.2 g/L MgCl_2_, and 0.1 g/L Ca(NO_3_)_2_ at pH7.5) (16), with lignocellulosic substrate being the sole carbon source. The liquid medium was supplemented with 0.1% vitamin solution (0.01 g/L biotin, 0.1 g/L riboflavin, 0.1 g/L nicotinic acid, 0.1 g/L thiamine, 0.1 g/L pyridoxal, 0.1 g/L folic acid) (18) and 100 mg/L cycloheximide. LCBM was sterilized by autoclaving (120°C, 30 min).

### Dilution-to-stimulation approach: development of the initial HD functional microbial community

The samples utilized in this study were collected from a naturally accumulated leaf litter located at the Northeast Agricultural University (Harbin, China). These leaves, which had undergone natural senescence and abscission during the autumn months, were gathered and arranged into an outdoor conical pile measuring 2 m in diameter and 1.3 m in height in October 2022. With the gradual rise in ambient temperatures during the ensuing spring, the leaves underwent progressive decomposition. In May 2023, a collection process was undertaken, wherein the desiccated outer layer of the pile was carefully removed to access and select semi-decomposed leaves from the central core for collection. Owing to their high cellulose content, these leaf litter serve as an excellent material for the enrichment and study of cellulose-degrading microorganisms. Three grams of sampled leaf litter and 10 g of sterile glass beads were added to 90 mL of MSM in a 250 mL conical flask to prepare the microbial inoculum. The flask was shaken at 250 rpm for 1 h at 30°C. Aliquots (250 μL) of the microbial suspension were added to 100 mL conical flasks in triplicate, containing 25 mL of LCBM with 1‰ vitamin solution. Culture conical flasks were incubated at 30°C and a rotation of 170 rpm under aerobic conditions. For the first two transfers (T1 and T2), aliquots of microbial suspension (250 μL) were transferred to 25 mL LCBM after 9 days of growth. From transfer 3 (T3) to T6, aliquots (50 μL) of the microbial suspension were transferred to 25 mL fresh LCBM and incubated under the same conditions for 4 days. Controls, including no substrate and no microbial source, were set up. After each batch culture, flasks were gently shaken, and solids were allowed to settle. The liquid fraction was collected; a portion of the liquid fraction was used to determine the optical density at 600 nm (OD_600nm_), and the remaining part was stored at −80°C for subsequent molecular biological investigation. For quantification of residual cellulose, the residual cellulose content was determined using a modified acid detergent fiber (ADF) method, based on Weimer et al. (19, 20). Briefly, 16.0 mL of acid detergent solution (2 mol/L H_2_SO_4_ containing 0.2% [wt/vol] cetyltrimethylammonium bromide, CTAB) was aseptically dispensed into culture bottles for each treatment group. The bottles were sealed with butyl rubber stoppers and sterilized at 121°C under pressurized steam for 45 min. Immediately after sterilization, the acid-insoluble residues were isolated via vacuum filtration using pre-dried quantitative filter papers (dried to constant weight at 105°C). To prevent cellulose recrystallization, the filtration system was maintained at >60°C during the process (21). Sequential washing was performed with three aliquots of preheated deionized water (80°C, total volume ≥200 mL). The filter papers retaining cellulose residues were transferred to pre-weighed aluminum crucibles and dried to constant weight in a forced-air oven at 105°C. Gravimetric analysis was conducted using an analytical balance. The substrate weight loss percentage was calculated as:


$$Weight\;loss\;(\%)=\left(\frac{W_{control}-W_{treatment}}{W_{control}}\right)\times 100$$

where $W_{control}$ and $W_{treatment}$ represent the dry weights of untreated (sterile control) and treated substrates, respectively.

### Dilution-to-extinction approach: selection of the LD functional community

Under sterile conditions, the HD functional microbial community (HD-T6, cultured for 5 days) was serially diluted with a 0.9% NaCl saline solution (pH 7.2). Briefly, 15 mL sterile centrifuge tubes were pre-filled with 9.0 mL of autoclaved saline (121°C, 20 min). A 1.0 mL aliquot of the original HD-T6 culture was transferred to the first tube (10⁻¹ dilution), vortex-mixed to ensure homogeneity (2,500 rpm, 30 s), and sequentially diluted by transferring 1.0 mL of the suspension to subsequent tubes containing 9.0 mL sterile saline. This process was repeated to generate a dilution series spanning 10⁻¹ to 10⁻³¹. Fresh sterile pipette tips were used at each dilution step to prevent cross-contamination.

From this full gradient, 13 critical dilutions (10⁻³, 10⁻⁵, 10⁻⁷, 10⁻⁹, 10⁻¹¹, 10⁻¹³, 10⁻¹⁹, 10⁻²¹, 10⁻²³, 10⁻²⁵, 10⁻²⁷, 10⁻²⁹, and 10⁻³¹) were selected for functional screening, targeting low-diversity yet metabolically active consortia. A 25 μL aliquot of each dilution was inoculated into 25 mL of LCBM medium supplemented with 0.1% (vol/vol) vitamin solution. Each dilution gradient was tested in triplicate biological replicates, with uninoculated sterile LCBM medium serving as a negative control. Cultures were incubated at 30°C for 5 days under continuous shaking (170 rpm). The microbial growth was quantified via optical density at 600 nm (OD_600_), and the percentage of substrate weight loss was also determined as described. At the 10⁻²¹ dilution level, a low-diversity yet effective functional microbial community was successfully established. To ensure functional stability, this consortium was subjected to five sequential subculturing cycles (5 d each, 0.1% [vol/vol] transfer inoculum into fresh LCBM medium), with consistent metabolic performance confirmed via ANOVA (*P* > 0.05 for degradation rate variation across cycles).

### Short-term experiment on cellulose degradation: 10-day analysis of HD/LD functional community

To examine the interaction between microbial community dynamics and cellulose degradation in HD and LD functional communities, we chose the stable HD of the transfer 6 (HD-T6) and the LD (dilution 10^−21^) of the transfer 5. To analyze replicates and changes over short time periods, three bottles each were collected at 24-hour intervals through a 10-day period (D1 to D10). Microbial suspension was collected, 10 mL from each bottle was taken to determine the enzyme activity related to cellulose degradation. Another 10 mL from each bottle was frozen for DNA extraction, metagenome, and metatranscription sequencing. The percentage of substrate weight loss was also determined as described in “Dilution-to-extinction approach: selection of the LD functional community,” above.

### Cellulose-degrading enzyme activity assays

The activity of cellulose degradation-related enzymes (Fpase and CMCase) was quantified by assessing the amount of reducing sugar produced by colorimetry (22). FPase and CMCase activities were determined using Whatman cellulose filter paper No. 1 (Whatman Ltd., England) and 1% CMC as substrates, respectively. The reaction mixtures were incubated in a water bath at 50°C for 30 min, and then the reactions were stopped by adding 3,5-dinitrosalicylic acid (DNS). Absorbance was recorded in 96-well plates at 540 nm. The β-glucosidase activities were determined using 4 mM p-nitrophenyl β-d-glucopyranoside (pNPG) as the substrate at 50°C for 30 min. The release of p-nitrophenol was determined by measuring absorbance at 410 nm. The unit of enzyme activity is expressed as the quantity of enzyme required to release 1 μg of product per milliliter substrate per minute under standard assay conditions.

### DNA extraction and high-throughput sequencing

DNA from the HD (HD-T1 to T6) and LD (LD-T5) functional microbial communities was extracted using the Bacterial DNA kit (Omega Bio-tek, Norcross, GA, U.S.), following the manufacturer’s instructions. After the DNA had been extracted, the bacterial 16S rRNA genes were amplified using the primers 338F (5′-ACTCCTACGGGAGGCAGCAG-3′) and 806R (5′-GGACTACHVGGGTWTCTAAT-3′) with a T100 Thermal Cycler (Bio-rad, USA). The PCR reaction mixture included 4 μL 5 × Fast Pfu buffer, 2 μL 2.5 mM dNTPs, 0.8 μL each primer (5 μM), 0.4 μL Fast Pfu polymerase, 10 ng of template DNA, and ddH_2_O to a final volume of 20 µL. PCR amplification cycling conditions were as follows: initial denaturation at 95°C for 3 min, followed by 27 cycles of denaturing at 95°C for 30 s, annealing at 55°C for 30 s and extension at 72°C for 45 s, and single extension at 72°C for 10 min, and end at 4°C. The PCR product was extracted from 2% agarose gel and purified using the PCR Clean-Up Kit (YuHua, Shanghai, China) according to the manufacturer’s instructions. The purified amplification products were analyzed on the Illumina platform at Majorbio (Shanghai, China).

### Metagenomic and metatranscriptional analysis

For the short-term experiment, samples from day 1 to day 10 of the HD and LD communities were collected for DNA and RNA extraction. The specific DNA and RNA extraction quality control, along with the subsequent steps for metagenomic and metatranscriptional analysis, are detailed in Text S1 in the supplemental material.

### *Cellulomonas* isolation and 16S rRNA gene sequencing

To validate the efficacy of the cellulose-degrading consortium, we attempted to isolate pivotal cellulolytic microbial members from the community. The stabilized LD consortium was subjected to serial dilution and plated onto selective medium plates containing pure cellulose filter paper as the sole carbon source. The medium consisted of 1 g/L (NH_4_)_2_SO_4_, 1 g/L KH_2_PO_4_, 0.7 g/L MgSO_4_.7H_2_O, 0.5 g/L NaCl, and Whatman cellulose filter paper No. 1 (Whatman Ltd., England) (one filter paper per culture dish). Individual colonies exhibiting cellulolytic potential were subsequently isolated and inoculated into liquid cellulose basal medium (LCBM) for further characterization. Single bacteria that play an important role in the community were successfully screened and classified into *Cellulomonas* using Sanger sequencing of the 16S rRNA gene. Short-term cellulose degradation experiments and cellulase activity assays of *Cellulomonas* were conducted according to the methodology described in “Short-term experiment on cellulose degradation: 10-day analysis of HD/LD functional community,” above.

### Statistical analysis

Statistical analyses were performed using JMP13 software (SAS Institute, Cary, NC, USA). A *P* < 0.05 was considered statistically significant. Charts were created using Origin 2022. The diversity and composition of microbial community were investigated using the I-Sanger platform (http://www. i-sanger.com/). Microbial community diversity was determined using the Shannon index in R software (version 4.2.1) with the “renyi” package (vegan) at the genus level to examine alpha diversity. Chao and Abundance-based Coverage Estimator (ACE) were also calculated using the “estimate” package in R software (version 4.2.1) to determine alpha diversity at the genus level. The abundance of OTUs by high-throughput sequencing (16S rRNA) was used for principal component analysis (PCA), which was performed using Canoco 5.0 (Biometris, Wageningen, the Netherlands) to explore the variability of the bacterial community composition between samples during the dilution-to-stimulation process. Based on the Bray-Curtis similarity distance matrix, non-metric multidimensional scaling (NMDS) and analysis of similarity (ANOSIM) were used to compare the similarity of the microbial community between different samples with R (v 4.2.1) vegan package. NMDS analysis was used to describe the difference in HD and LD microbial community composition. ANOSIM analysis was used to test whether the difference between the HD and LD communities was significantly greater than the difference within the group. Stepwise regression and path models were done to study the direct and indirect effects of microorganisms related to cellulose hydrolysis on cellulase activity (FPase, CMCase, and β-glucosidase) and substrate degradation using Data Processing System (DPS) software. Microbial co-occurrence network analysis was performed based on Spearman in R version 4.0.3 using the “psych” package (23). Correlation coefficients greater than 0.8 with a corresponding *P* < 0.05 were considered statistically robust and were included to generate the network. The network structure was visualized using the Gephi 0.9.7 program.

## RESULTS AND DISCUSSION

### Initial enrichment of a HD functional microbial community by the dilution-to-stimulation approach

**Fig 1:**
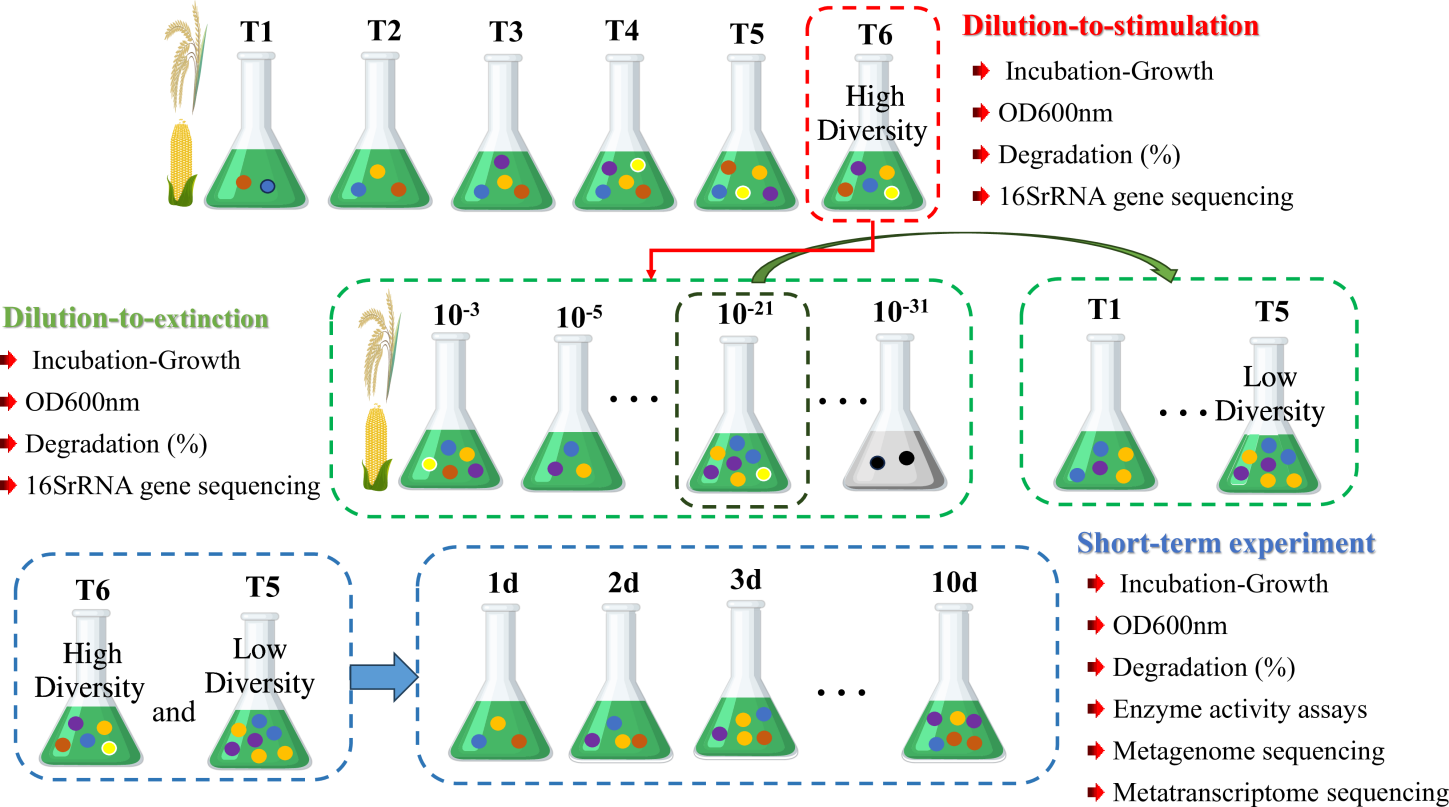
Schematic representation of the combined top-down enrichment strategy (i.e., dilution-to-stimulation coupled to dilution-to-extinction) used to select the high-diversity and low-diversity effective functional microbial community.

**Fig 2:**
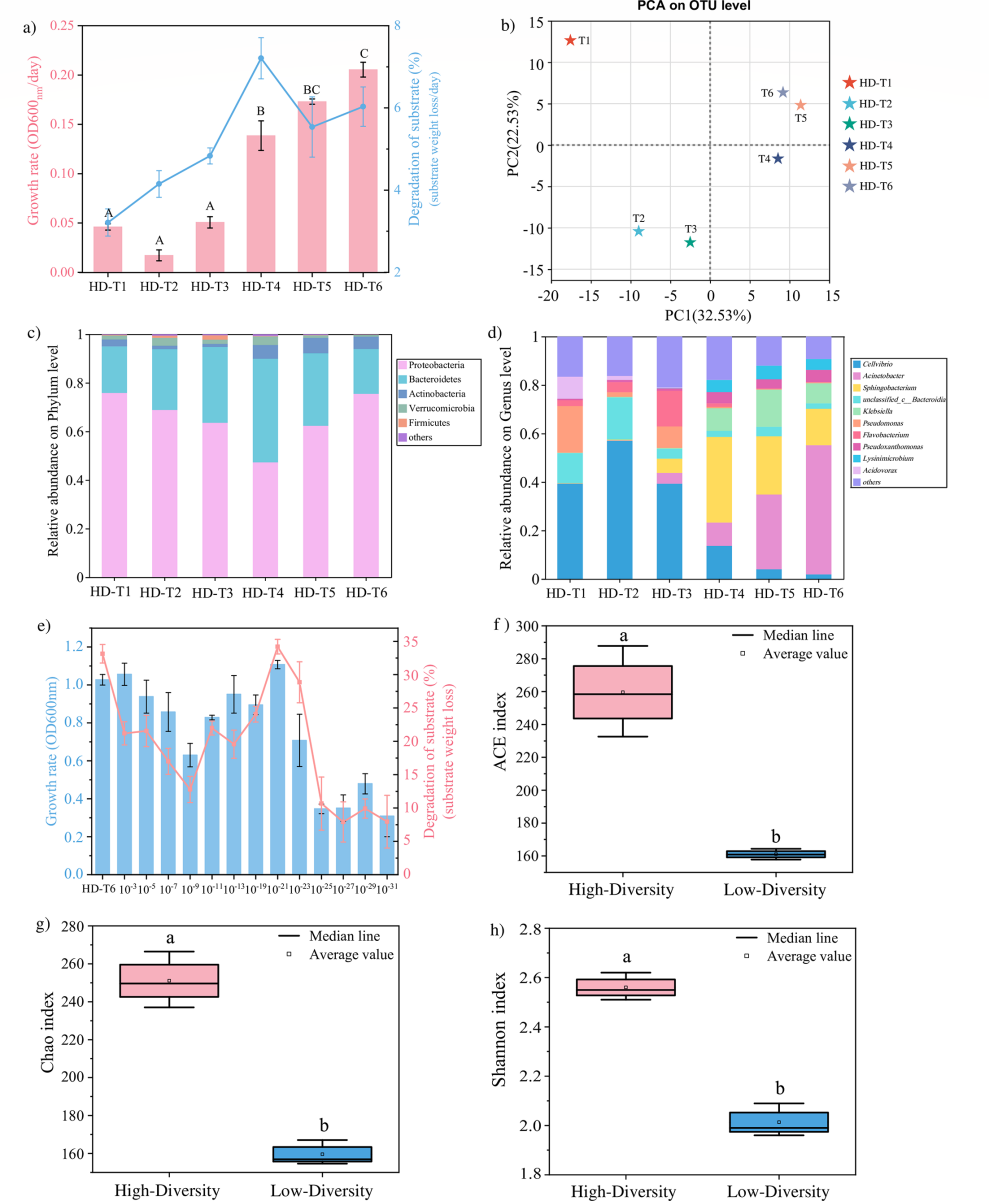
Dilution-to-stimulation/extinction approach used for the selection of the high-diversity and low-diversity effective functional microbial community. (**a**) Microbial growth rate (OD_600nm_ per day) and degradation of substrate (substrate weight loss/day) along the sequential transfers during the process of dilution-to-stimulation. (**b**) Principal components analysis (PCA) of microbial community composition along the sequential transfers during the process of dilution-to-stimulation. (**c**) The composition of microbial community at phylum along the sequential transfers during the process of dilution-to-stimulation. (**d**) The composition of microbial community at genus along the sequential transfers during the process of dilution-to-stimulation. (**e**) Microbial growth rate (OD_600nm_) and degradation of substrate (substrate weight loss) under different dilutions during the process of dilution-to-extinction. (**f–h**) Shannon index (ACE, Chao, Shannon) value within high-diversity and low-diversity communities. Different letters indicate significant differences among mean values (*P* < 0.05).

The dilution-to-stimulation approach was employed to enrich the initial HD microbial community with high lignocellulose degradation potential. The litter-derived community used a substrate composed of maize straw and rice straw (Fig. 1). During the enrichment process, the incubation time was reduced from 9 days in transfers 1 and 2 (HD-T1 and T2) to 4 days after the last transfers (HD-T3 to T6). The observed differences in incubation times across the transfers were statistically significant (*P* < 0.05) (Fig. 2a), suggesting a higher growth rate and consortium stabilization after transfer 4 (HD-T4). Moreover, the average weight loss per day of the substrate exhibited an increasing trend during the transfers, increasing from 3.21% ± 0.33% in HD-T1 to a maximum 7.21% ± 0.50% in HD-T4 (Fig. 2a). These results showed that the initial HD microbial community became more effective in degrading lignocellulose substrate during sequential transfers. After T4, the substrate weight loss reached a plateau (Fig. 2a), which indicated that an HD community capable of stably degrading cellulose was successfully enriched.

To elucidate changes in community structure across the transfers, 16S rRNA gene sequencing was performed. The PCA was employed to assess the similarities and differences of the microbial community structures across transfers (Fig. 2b). There were similarities in the structure of microbial communities within the T4–T6 and differences in the structure among the HD-T1 to T3 (Fig. 2b), which indicated that the microbial community composition stabilized during the transfer. At the phylum level, Proteobacteria, Bacteroidetes, and Actinobacteria represented 47.28–75.85%, 18.45–42.62%, and 1.27–6.33% of the relative abundance during the transfers, respectively (Fig. 2c). These frequently appear as dominant players in bacterial communities involved in the degradation of lignocellulose substrates (24). At the genus level, *Cellvibrio* was the dominant genus during HD-T1 to T3. However, the stabilized consortium (HD-T4 to T6) contained high proportions of species belonging to the genus *Acinetobacter*, followed by the *Sphingobacterium* and *Klebsiella* (Fig. 2d). *Sphingobacterium* and *Klebsiella* play important roles during the cellulose degradation.

### Selection and identification of a LD functional microbial community by the dilution-to-extinction approach

The reduction of microbial diversity and complexity using the dilution-to-extinction method has been widely applied (25, 26). In this study, an LD-efficient functional microbial community was achieved by dilution to extinction of the initial HD functional microbial community (obtained at T6, HD-T6). To reduce the microbial diversity within this community, serial dilutions ranging from 10^−3^ to 10^−31^ were performed (Fig. 2e). The degradation ability of substrate does not increase with dilution, which was due to stochastic processes caused by extinction leading to different microbial community compositions (16). The most effective but lowest microbial community was found in the 10^−21^ dilution with high cell densities (OD _600nm_), and a high value of substrate weight loss was obtained in the 10^−21^ (Fig. 2e). To ensure the stability of the microbial function, this LD functional microbial community (10–19, 21, 22) was subsequently transferred five times (LD-T1 to T5). After five transfers, the community maintained the high substrate weight loss value (LD-T5: 31.65%) (Fig. S1). These results indicated that the LD community was a promising candidate for cellulose degradation with the potential for practical application. The bacterial 16S rRNA high-throughput sequencing was done with the HD (HD-T6) and LD (LD-T5) functional communities. The alpha diversity estimators of three indices (ACE, Chao, and Shannon index) of HD and LD microbial communities are shown in Fig. 2f through h. The ACE and Chao indices represent community richness, and the Shannon indices represent community diversity. LD functional community (ACE, 161.03; Chao, 159.54; Shannon, 2.01) exhibited significantly lower richness and diversity than HD functional community (ACE, 259.59; Chao, 251.06; Shannon, 2.56) (Fig. 2f through h). These results indicated that dilution-to-extinction successfully reduced species richness and diversity, and a microbial community with LD but stable cellulose degradation function has been successfully obtained in this study.

### Interaction of functional and key microbes of HD and LD communities during cellulose degradation

#### Lignocellulolytic capacity and the cellulolytic enzyme activity of HD and LD microbial communities

**Fig 3:**
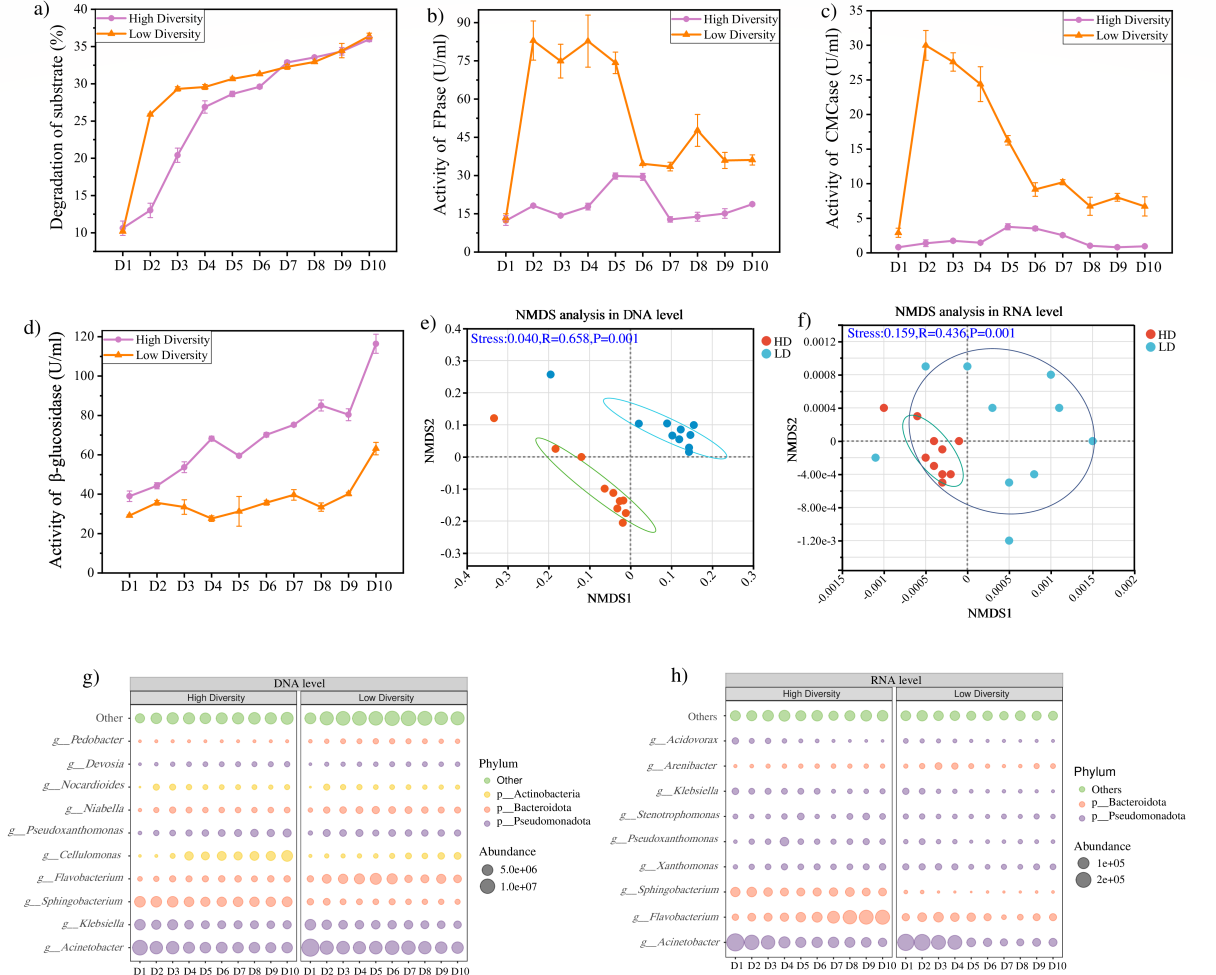
Degradation of substrate, activity of key cellulolytic enzyme, and composition of microbial community during lignocellulose substrate degradation in both high-diversity and low-diversity communities. (**a**) Degradation of substrate. (**b–d**) Activity of key cellulolytic enzyme (FPase, CMCase, β-glucosidase) (**e**) Non-metric multidimensional scaling (NMDS) of microbial community in the metagenome. (**f**) Non-metric multidimensional scaling (NMDS) of microbial community in the metatranscription. (**g**) Composition of microbial community in the metagenome. (**h**) Composition of microbial community in the metatranscription.

To investigate the community dynamics and interactions among the members of the community, a 10-day short-term experiment on cellulose degradation by HD and LD microbial communities (HD-T6 and LD-T5) was performed. The degradation of the substrate is shown in Fig. 3a. In the HD community, substrate degradation mainly occurred in the first 4 days, reaching 26.89% on day 4. By contrast, in the LD community, the substrate degradation reached only 29.31% on the 3rd day and subsequently slowed down. These results indicated that the main period of substrate degradation by both LD and HD communities occurred during the earlier stage of cultivation, which was consistent with other studies (27, 28). In both communities, substrate degradation rates remained stable from days 7 to 10 (Fig. 3a). On day 10, the degradation of substrate in the LD community (36.42%) was slightly higher than the HD community (35.98%) (Fig. 3a), suggesting that LD communities accelerate the cellulose degradation process and have a greater capacity for cellulose degradation. This may be due to cooperation between functional microorganisms in the LD community and the functional redundancy theory of microbial communities.

The changes in the activities of key cellulose-degrading enzymes during the short-term experiment revealed distinct dynamic patterns between the HD and LD communities (Fig. 3b through d). Filter paper enzyme (FPase) activity serves as a principal parameter for assessing the practical application performance of cellulase, as it accurately reflects the synergistic effect of enzymatic hydrolysis on natural cellulose (29). CMCase, which is responsible for endoglucanase activity, plays a crucial role in the degradation of cellulose (20). The dynamic patterns of FPase and CMCase activity were markedly distinct between the HD and LD communities (Fig. 3b and c). The LD community showed a robust and fast enzymatic reaction, with FPase and CMCase activities dramatically increasing and peaking on the second day (FPase: 13.39–82.97 U/mL; CMCase: 2.91–29.99 U/mL), explaining the rapid substrate degradation in LD communities during the 1–2 days (Fig. 3a). Jiang et al. developed a minimal synthetic community comprising only four bacterial strains for lignocellulose degradation, which achieved a high cellulase activity of 103.16 U/mL and substantial substrate degradation within 3 days (30). Subsequently, the FPase activity of the LD community remained stable until day 5 and then showed a downward trend from days 6 to 10 (34.60–36.15 U/mL), when the substrate degradation tended to stabilize. The activity of CMCase rapidly decreased as the degradation rate slowed down (days 2–6) and then stabilized.

Compared to the LD community, the activity of FPase and CMCase in the HD community remained at a lower level in the initial stage and began to increase substantially on day 4. The activity of FPase and CMCase remained high between days 4 and 6, then gradually decreased from day 6 with the depletion of substrate. The peak activity of the HD community (FPase: 29.82 U/mL; CMCase: 3.77 U/mL) was obviously lower than that of the LD community (FPase: 82.97 U/mL; CMCase: 29.99 U/mL). This observation may be attributed to the fact that the LD community can more effectively degrade lignocellulosic substrates (Fig. 3a), which underpins the synergistic effect of enzyme activities on cellulosic waste (31). Higher cellulolytic enzyme activities cause cellulolytic substrate decomposition (32). The observed activity peak of the HD community is consistent with the reported maximum cellulase levels in enriched lignocellulosic communities from complex environments. The thermophilic lignocellulolytic consortium TMC7 exhibited a maximum exoglucanase activity of 5.51 U/mL on day 6 during the alkali-treated corn stover degradation (27). These studies indicate different decomposition strategies between HD and LD communities. The LD community primarily relies on the rapid and copious production of hydrolytic enzymes in the early phase. In contrast, the HD community, due to greater functional redundancy and potential for complex interspecies interactions, may require a longer period to coordinate its enzymatic machinery, resulting in delayed and reduced peak activity.

β-Glucosidase, a crucial enzyme in the final stages of cellulose degradation, facilitates the conversion of cellobiose into glucose and is an important enzyme during cellulose degradation (33). In this study, the activity of β-glucosidase showed an increasing trend in LD and HD communities during cellulose degradation (Fig. 3d), which is due to the accumulation of a large amount of cellobiose during cellulose degradation. However, compared with endoglucanase and exoglucanase, the activity profile of β-glucosidase (BGL) differs substantially from the trend of cellulose degradation efficiency. Endoglucanase and exoglucanase directly participate in the initial depolymerization process of cellulose (34), so their activity dynamics are basically consistent with the cellulose degradation rate. In contrast, BGL does not directly act on cellulose substrates. Its catalytic activity is mainly limited by the supply of intermediate products produced by the hydrolysis of upstream endo/exo-glucanases, such as cellobiose (23). In addition, BGL is also susceptible to feedback inhibition from the final product (glucose) of cellulose decomposition (35). Therefore, BGL activity cannot be used as an indicator for predicting the overall degradation efficiency of cellulose. Compared with LD, the higher BGL activity observed in the HD community is inconsistent with the substrate degradation trends of the two communities, but this phenomenon provides clear support for the above-mentioned views.

#### Composition of HD and LD microbial communities

Metagenomic and metatranscription sequencing were performed to investigate changes in the microbial community structure of LD and HD communities during the lignocellulosic substrate degradation. The microbial community composition differed significantly between the LD and HD communities according to NMDS analysis (Fig. 3e and f), which is due to stochastic reassembly of microbes caused by dilution-extinction, and the initial microbial community was altered due to the serial dilution process (14, 36). Typically, a reduction in microbial biodiversity may result in a decline in microbial functions (37, 38). Nevertheless, the degradation of the lignocellulosic substrate was slightly increased but did not decline in the LD community in our study. The NMDS analysis also suggested that significant alterations in community composition did not impact the degradation of lignocellulosic substrate. The concept of functional redundancy can elucidate these results, and different microorganisms might possess similar functions but perform optimally under diverse conditions, substituting for each other to maintain ecosystem stability (39).

The changes in microbial community composition at the DNA and RNA levels are shown in Fig. 3g and h. At the phylum level, Pseudomonadota and Bacteroidota were identified as the dominant phyla in both the metagenome (Fig. 3g) and metatranscriptome (Fig. 3h) of HD and LD communities. These two phyla were also reported as the dominant phyla in other lignocellulosic degradation environments (24, 35). At the genus level, *Acinetobacter*, *Klebsiella*, *Sphingobacterium*, and *Flavobacterium* accounted for a substantial proportion of the HD and LD communities based on metagenomic (DNA-level) data, indicating that these highly abundant taxa did not become extinct and maintained high abundance during the dilution-to-extinction process. In contrast, as reported by Miao et al., rare species were more vulnerable to this process (26). At the RNA level, *Acinetobacter*, *Flavobacterium*, and *Sphingobacterium* continued to dominate in both communities, suggesting that these genera not only persisted but also maintained high transcriptional activity.

#### Composition and putative metabolic pathway of cellulose-degrading microbial community

**Fig 4:**
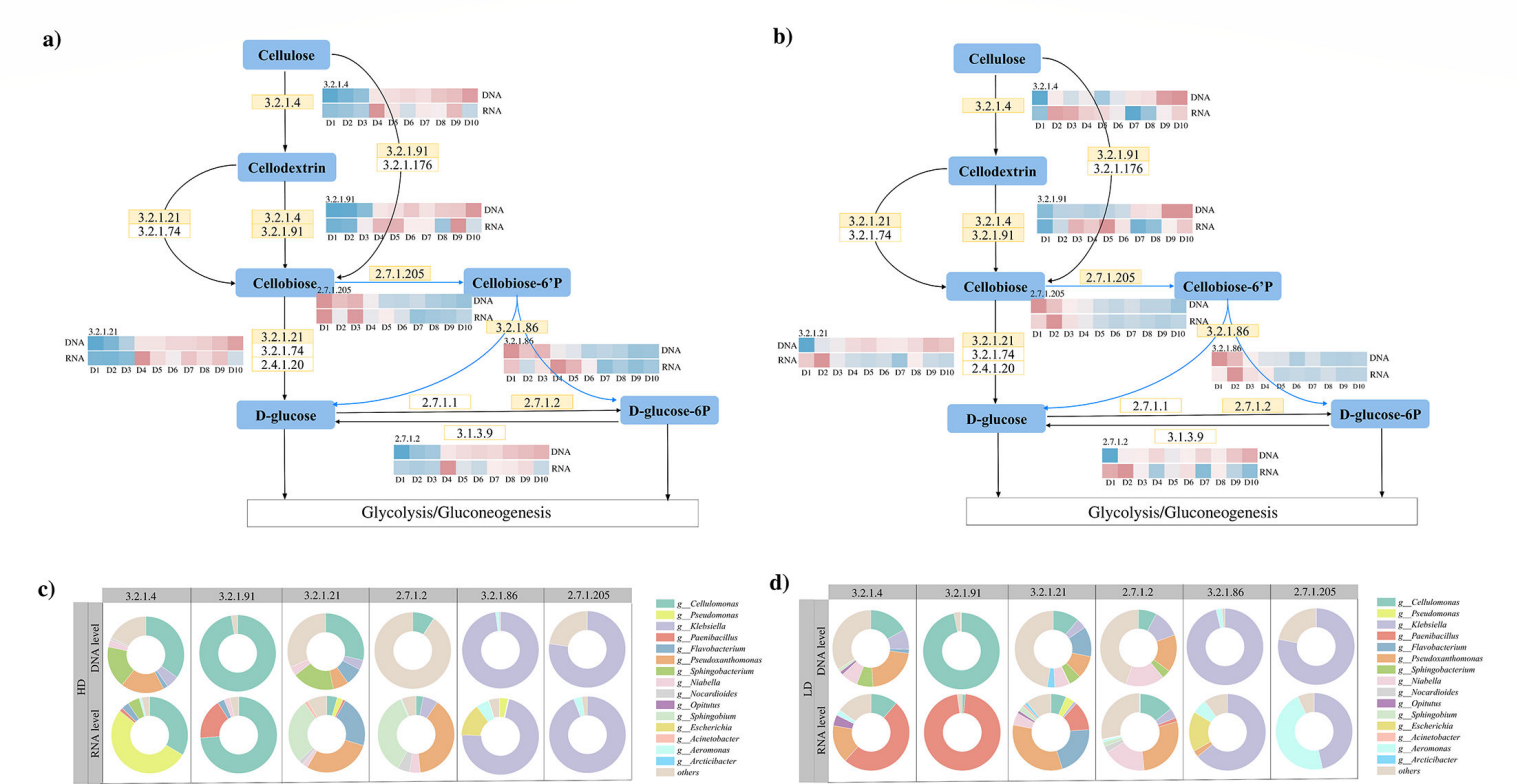
The metabolic pathway relating to cellulolytic catabolism in both high-diversity (**a**) and low-diversity communities (**b**). Metabolites were exhibited in blue boxes. Arrows indicate both the pathways and directions of metabolism. The numbers in orange boxes refer to the EC number of the enzymes. Solid represents the presence of this enzyme in the community involved in this pathway, and hollow represents its absence. The heatmap depicts the abundance and transcript levels of functional genes involved in cellulose degradation, with blue and red hues representing lower and higher values, respectively. The microbial community composition of functional genes in metagenomics and metatranscriptomics across high-diversity (**c**) and low-diversity (**d**) microbial communities.

The role of functional microorganisms is important during cellulose degradation, and the processes that drive the synthesis and decomposition of cellulose were inherently determined by bioenzymes (4). To elucidate the synergistic mechanisms of the LD and HD communities in cellulose degradation, we investigated their metabolic potential by reconstructing pathways based on KEGG analysis and profiling the dynamics of key cellulase genes at both DNA and RNA levels. The enzyme genes related to cellulose metabolism, annotated by KEGG in metagenome and metatranscriptome, are listed in Tables S1 and S2. Both HD and LD communities can secrete and establish complex enzyme systems, including exoglucosidase and endoglucosidase (Fig. 4a and b), which is the reason why both communities can effectively degrade cellulose.

In the first step, cellulose was first hydrolyzed into cellodextrin and cellobiose by endo-1,4-β-glucanase (EC 3.2.1.4), exo-1,4-β-glucanase (EC 3.2.1.91), and cellulose 1,4-beta-cellobiosidase (reducing end) (EC 3.2.1.176) (Fig. 4a and b) (40). The genes encoding endo-1,4-β-glucanase (EC 3.2.1.4) and exo-1,4-β-glucanase (EC 3.2.1.91) were identified in both communities and exhibited transcriptional activity. However, genes for cellulose 1,4-beta-cellobiosidase (EC 3.2.1.176) were not identified in the metagenome and metatranscriptome. In the next step, except for endo-1,4-β-D-glucanase, which hydrolyzed cellodextrin into cellobiose, cellodextrin could also be hydrolyzed into cellobiose and D-glucose by β-glucosidase (EC 3.2.1.21) and exo-β−1,4-glucosidase (EC 3.2.1.74) (40). While the encoding capacity for β-glucosidase was present and actively transcribed in both communities, the genes for exo-β−1,4-glucosidase (EC 3.2.1.74) were not detected in either the metagenome or metatranscriptome of the communities. Finally, previous research has indicated that cellobiose metabolism involves mainly three pathways: (i) β-glucosidase (EC 3.2.1.21) mediated hydrolytic processes; (ii) 6-P-β-glucosidase (EC 3.2.1.86) mediated ATP-dependent hydrolytic processes that enable simultaneous translocation and phosphorylation of cellobiose; and (iii) cellobiose phosphorylases (EC 2.4.1.20) mediate inorganic phosphate-dependent phosphorolytic process (41). The pathway (i) and pathway (ii) of cellobiose metabolism coexist in both HD and LD communities, but pathway (iii) was not found (Fig. 4a and b). In brief, in both HD and LD communities, cellobiose can be completely hydrolyzed into glucose via a one-step reaction catalyzed by β-glucosidase (EC 3.2.1.21). Additionally, cellobiose can be enzymatically converted into glucose through a sequential reaction involving cellobiose phosphotransferase system (PTS) (EC 2.7.1.205), 6-phospho-β-glucosidase (EC 3.2.1.86), and glucokinase (EC 2.7.1.2).

The production of enzymes by microorganisms at specific periods also served as a crucial indicator of cellulose degradation. The abundance and transcription of functional genes were analyzed during cellulose degradation using the metagenome and metatranscriptome analyses. In the HD community, the transcription of genes encoding enzymes associated with the cellulose hydrolysis pathway (EC 3.2.1.4, EC 3.2.1.91) exhibited an increasing trend after 3 and 2 days (Fig. 4a). Meanwhile, the transcription of these functional genes rapidly increased on the second day in the LD community (Fig. 4b), which further explains the accelerated cellulose degradation by the LD communities (Fig. 3a through d). Interestingly, a significant positive correlation was observed between the genomic abundance and transcript levels of enzymes (EC 3.2.1.4, EC 3.2.1.91) responsible for cellulose chain deconstruction in HD communities, which does not exist in LD communities (Fig. S2). This suggests that high diversity leads to stronger community functional redundancy and robustness (42, 43), and genetic potential remains stable in the changing environment during the cellulose degradation process, which is consistent with biological insurance theory (44).

The composition of cellulolytic microbial communities was also observed in HD and LD communities. At the DNA level, *Cellulomonas* (Actinobacteria) is the main genus in the cellulose hydrolysis pathway in the HD community, followed by *Sphingobacterium* (Bacteroidetes) and *Pseudoxanthomonas* (Pseudomonadota) (Fig. 4c). And in the LD community, *Cellulomonas* (Actinobacteria) and *Pseudoxanthomonas* (Pseudomonadota) are the dominant genera in the cellulose hydrolysis pathway (Fig. 4d). It is worth noting that some functional genes with low abundance in DNA showed high transcriptional activity during cellulose degradation (45). *Pseudomonas* (Pseudomonadota) and *Paenibacillus* (Bacillota) are representative of this scenario in the HD and LD communities, respectively.

#### Key functional microorganism affecting cellulose hydrolysis

**Fig 5:**
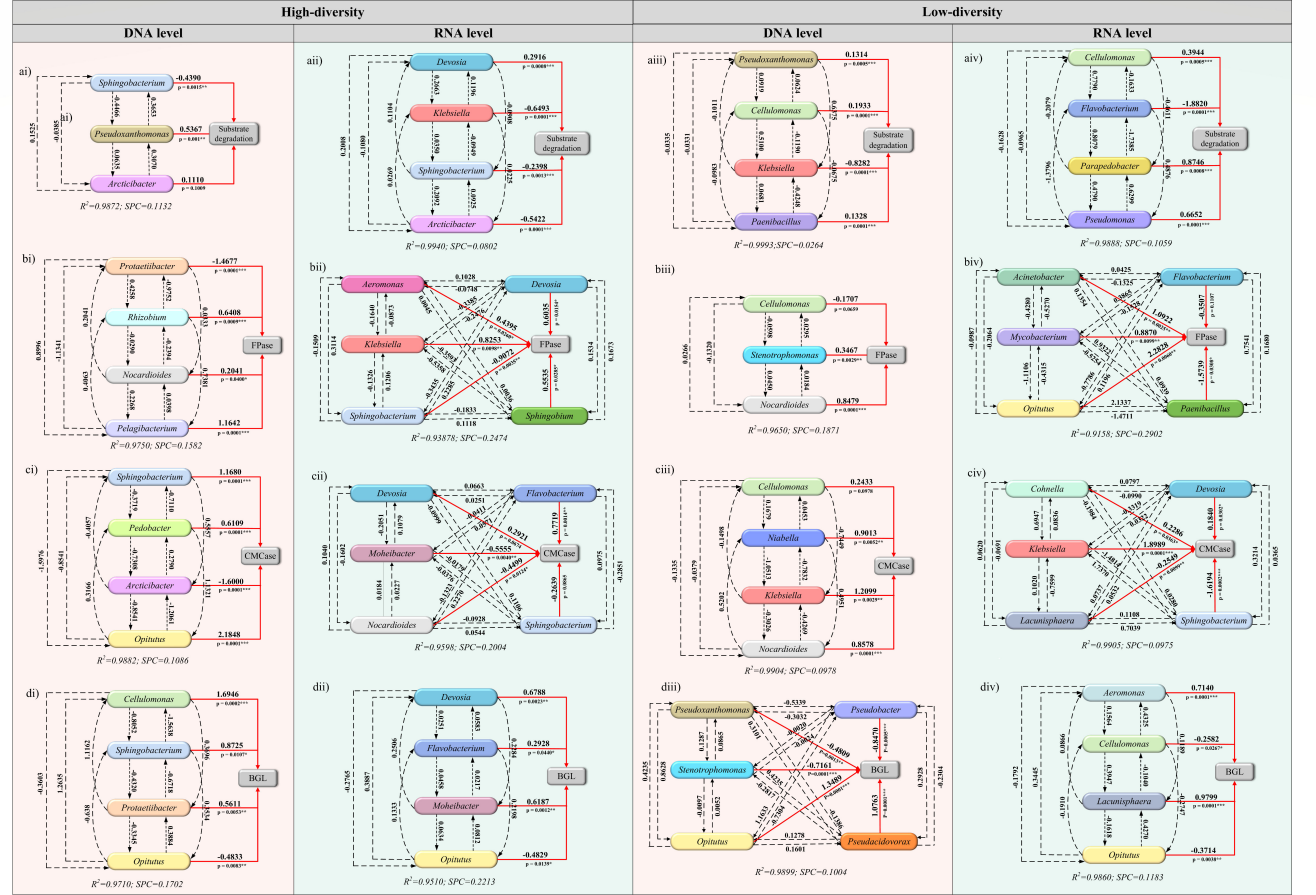
Path diagram estimating the direct and indirect effects of functional microorganisms related to cellulose hydrolysis on substrate degradation (ai–aiv), FPase activity (bi–biv), CMCase activity (ci–civ), and β-glucosidase (di–div) in high-diversity community (ai–di and aii–dii) and low-diversity community (aiii–diii and aiv–div) at the DNA and RNA level. Arrows designate the direction of causality, and the numbers (path coefficient, λ) adjacent to arrows represent the degree of direct (solid line) and indirect (dotted line) effects. *R*^2^: Determination coefficient; SPC: Surplus path coefficient. *P*: *0.01 ≤ *P* < 0.05, **0.001 ≤ *P* < 0.01, ***P < 0.001.

To quantify the link between functional community and cellulose degradation, and figure out the key functional communities associated with cellulose hydrolysis in the HD and LD communities, path analysis at the DNA and RNA level was performed to provide a quantitative link between cellulose degradation related indicators (dependent variable) and cellulose hydrolysis-related microorganism genera (independent variable). The high *R*^2^ and significant *P* (*P* < 0.05) values (Table 1), along with low surplus path coefficient (SPC, Fig. 5) for the examined factors observed in HD and LD communities, indicate strong linear and quantitative associations between the key functional microorganism genera and cellulose degradation.

**TABLE 1:** Stepwise regression model with substrate degradation and key cellulolytic enzyme activity as dependent variables and functional microorganisms related to cellulose hydrolysis as independent variables (*n* = 10)

Treatment	Standardization regression equations	***R*** ^**2**^	***P***-value^*a*^
High diversity			
DNA level	Substrate degradation = 0.4400 – 0.0016 *Sphingobacterium* + 0.0008 *Sphingobacterium* + 0.0024 *Arcticibacter*	0.9936	0.0001^***^
	FPase = 1.7846 – 2.5402 *Protaetiibacter* + 2.0446 *Rhizobium* + 0.2317 *Nocardioides* + 3.5730 *Pelagibacterium*	0.9874	0.0003^***^
	CMCase = −10.4472 + 0.0543 *Sphingobacterium* + 0.3009 *Pedobacter* – 0.4396 *Arcticibacter* + 0.859502331 *Opitutus*	0.9941	0.0001^***^
	BGL = −167.3688 + 0.1590 *Cellulomonas* + 0.8258 *Sphingobacterium* + 3.3888 *Protaetiibacter* − 3.865425088 *Opitutus*	0.9854	0.0005^***^
RNA level	Substrate degradation = 0.4221 + 0.0120 *Devosia* – 0.0012 *Klebsiella* – 0.0070 *Sphingobacterium* – 0.0612 *Arcticibacter*	0.9968	0.0001^***^
	FPase = 11.6444 + 0.3563 *Aeromonas* + 1.778 *Devosia* + 0.1064 *Klebsiella* – 1.8826 *Sphingobacterium* +0.0659 *Sphingobium*	0.9689	0.0154^*^
	CMCase = 1.5619 + 0.1477 *Devosia* + 0.0186 *Flavobacterium* – 0.0708 *Moheibacter* – 0.0196 *Nocardioides* – 0.0940 *Sphingobacterium*	0.9797	0.0068^**^
	BGL = 32.8815 + 6.9792 *Devosia* + 0.1435 *Flavobacterium* + 1.6027 *Moheibacter* – 11.9220 *Opitutus*	0.9752	0.0018^**^
Low diversity			
DNA level	Substrate degradation = 0.4510 + 0.0003 *Pseudoxanthomonas* + 0.0002 *Cellulomonas* – 0.0048 *Klebsiella* + 0.0009 *Paenibacillus*	0.9997	0.0001^***^
	FPase = −52.1022 – 0.0515 *Cellulomonas* + 5.3687 *Stenotrophomonas* + 4.8345 *Nocardioides*	0.9823	0.0001^***^
	CMCase = −82.5225 + 0.0286 *Cellulomonas* + 0.6985 *Niabella* + 0.9384 *Klebsiella* + 1.9138 *Nocardioides*	0.9952	0.0001^***^
	BGL = 83.5138-0.1521 *Pseudoxanthomonas* – 4.4857 *Stenotrophomonas* + 2.1167 *Opitutus* – 4.0282 *Pseudobacter* + 6.2014 *Pseudacidovorax*	0.9950	0.0004^***^
RNA level	Substrate degradation = 0.3201 + 0.0009 *Cellulomonas* – 0.0040 *Flavobacterium* + 001878 *Parapedobacter* + 0.0035 *Pseudomonas*	0.9944	0.0001^***^
	FPase =20.2773 + 4.2788 *Acinetobacter* – 3.2738 *Flavobacterium* + 4.0199 *Mycobacterium* + 4.1007 *Opitutus* – 0.6077 *Paenibacillus*	0.9570	0.0285^*^
	CMCase = 13.5446 + 0.7401 *Cohnella* – 0.8695 *Devosia + 0*.4845 *Klebsiella* + 0.9312 *Lacunisphaera – 3.8934 Sphingobacterium*	0.9952	0.0004^***^
	BGL = 44.1229 – 1.1314 *Aeromonas* – 0.0835 *Cellulomonas* + 3.7005 *Lacunisphaera* – 0.6808 *Opitutus*	0.9930	0.0001^***^

^*a*^ *, significance at 0.01 *≤ P* < 0.05; **, significance at 0.001 *≤ P* < 0.01; ***, significance at *P* < 0.001.

At the DNA level, the key functional groups regulating substrate degradation in the HD community were *Sphingobacterium*, *Pseudoxanthomonas*, and *Arcticibacter*. Among them, *Sphingobacterium* (λ = −0.4490**) and *Pseudoxanthomonas* (λ = 0.5367**) directly affected substrate degradation (Fig. 5ai). This is consistent with the finding that cellulolytic microorganisms from these two genera constitute a considerable proportion of the HD community and possess genes encoding endoglucanase and β-glucosidase (Fig. 4c). Furthermore, *Sphingobacterium* was identified as the key group regulating CMCase (λ = 1.1680***) and β-glucosidase (λ = 0.8725*) activities (Fig. 5ci and di). At the RNA level, *Sphingobacterium* also directly influenced substrate degradation (λ = −0.2398***), FPase (λ = 0.5535*), and CMCase (λ = 0.2639) activities (Fig. 5aii through cii). Collectively, these results suggest that *Sphingobacterium* is a key genus mediating cellulose degradation in the HD community (Fig. 5a), which is consistent with previous reports on its efficient lignocellulosic degradation capabilities (46). Notably, *Devosia* also directly affected substrate degradation (λ = 0.2916*), FPase (λ = 0.6035*), CMCase (λ = 0.2921), and BGL (λ = 0.6788) activities (Fig. 5aii through dii). Although *Devosia* maintains low abundance in the metatranscriptome, it may occupy a critical ecological niche in the cellulose degradation process of the HD community, as its positive correlation with lignocellulose degradation has been widely documented (47, 48).

In the LD community, although dilution-extinction altered the key functional microbial groups affecting substrate degradation, a close correlation between genera *Pseudoxanthomonas* (λ = 0.1314***) and substrate degradation was also observed at the DNA level (Fig. 5aiii). This result is consistent with the HD community (Fig. 5ai). *Pseudoxanthomonas* has been identified as a key genus in some cellulose-degrading consortia and plays a significant role by boosting cellulose degradation (44, 45). Besides, *Paenibacillus* (λ = 0.1328***), *Cellulomonas* (λ = 0.1933***)*,* and *Klebsiella* (λ = −0.8282***) also had a direct effect on the substrate degradation at the DNA level (Fig. 5aiii). Some species belonging to the genus *Paenibacillus* have an advantageous position in lignocellulose-rich environments (2), possessing effective lignolytic and cellulosytic enzymes (49). The genus *Cellulomonas* includes some efficient cellulose degraders, as has been previously reported (50), and it is used for industrial cellulase production (51). For the enzymes related to cellulose degradation, *Cellulomonas* (FPase: λ = −0.1707; CMCase: λ = 0.2433) and *Stenotrophomonas* (FPase: λ = 0.3467***; β-glucosidase: λ = −0.7161***) showed direct impact on cellulolytic enzymes at the DNA level. Previous research by Liu et al. (52) has highlighted *Stenotrophomonas* as a key player in lignocellulose degradation. At the RNA level, *Cellulomonas* also directly influenced substrate degradation (λ = −0.3944***) and CMCase (λ = −0.2582) activities (Fig. 5aiv and div). These results indicated that *Cellulomonas* was the vital functional genus during cellulose degradation in the LD community. *Cellulomonas* have the full suite of genes necessary to deconstruct cellulose, including exoglucanase, endoglucanase, and β-glucosidase (Fig. 4c and d).

#### Co-occurrence pattern of cellulolytic microbial community

**Fig 6:**
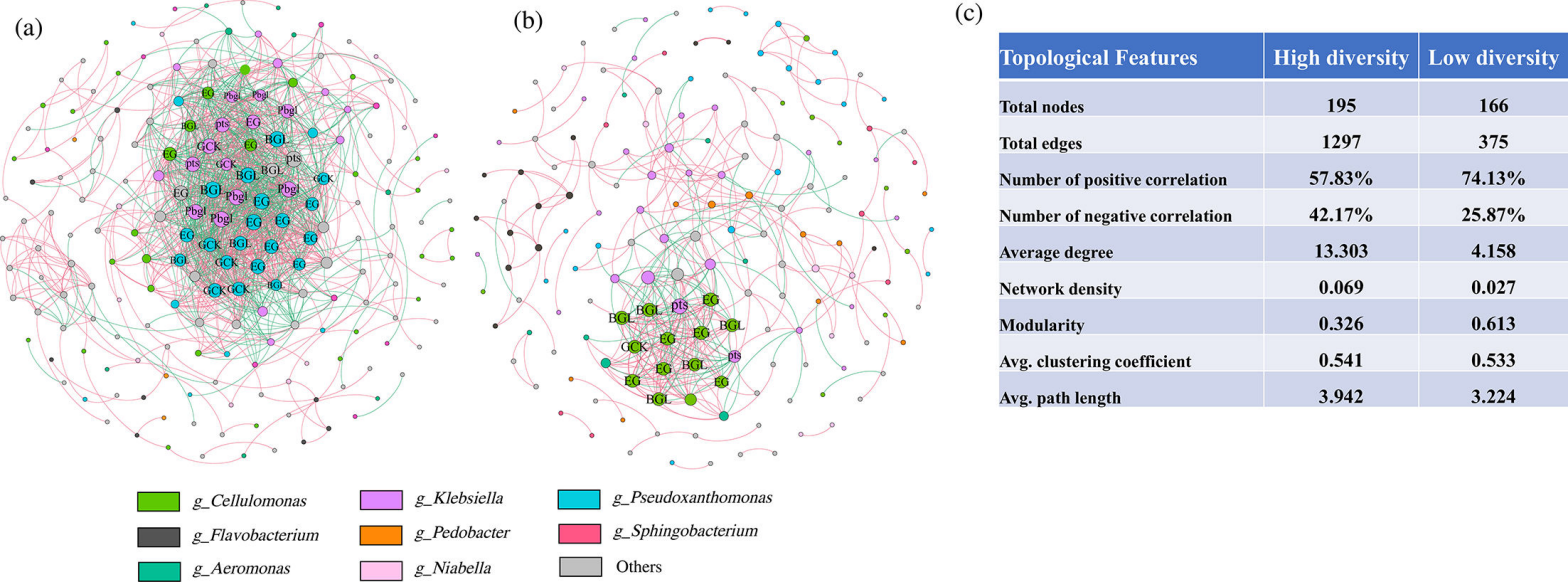
Co-occurrence network analysis of cellulolytic microbial communities (**a**) in the high-diversity community and (**b**) in the low-diversity community. (**c**) Key topological features of networks. The nodes were colored by taxonomy at the genus level. Each node size represents its degree. Red lines denote significantly positive correlation, and green lines represent significantly negative correlation (*P* < 0.05). EG: endoglucanase; BGL: β-glucosidase; GCK: glucokinase; pts: D-cellobiose PTS permease; Pbgl: 6-phospho-β-glucosidase.

To investigate the potential interactions of microbial communities related to cellulose hydrolysis in the HD and LD communities, the co-occurrence networks of the functional microbial community were constructed using the random matrix theory (Fig. 6a and b). The construction of the symbiotic networks used the same thresholds (*r* > 0.8, *P* < 0.05), and the key topological features are summarized in Fig. 6c. Overall, network complexity varied considerably between the HD and LD communities. The network consisted of 195 nodes and 1,297 edges in the HD community, compared to 166 nodes and 375 edges in the LD community (Fig. 6c). Additionally, the network density of the HD community (0.069) is obviously higher in the LD community (0.027) (Fig. 6c). These results showed that reducing community diversity can decrease the complexity of the networks and make the network simpler. Previous research has demonstrated that network complexity increased with microbial diversity (53, 54). The modularity value in the LD community (0.613) was greater than 0.4, demonstrating that the networks of the LD community had a small-world and modular structure (55), with a more organized structure among microorganisms. Moreover, the proportion of positive correlations was 74.13% in the LD community, significantly higher than in the HD community (57.83%) (Fig. 6c). These positive interactions in the co-occurrence network contribute to the mutualism and cooperation of the LD community, and this is one of the reasons why the substrate degradation rate was not reduced but rather increased in the LD community.

Nodes in the network exhibiting high connectivity are often identified as the keystone members, which play an integral role in stabilizing the structure and function of the ecosystem (55, 56). Keystone taxa changed as the diversity of microbial communities decreased (Fig. 6a and b). In the HD community, the keystone taxa belonged to *Pseudoxanthomonas* and *Klebsiella*. While in the LD community, keystone taxa belonged to *Cellulomonas* and *Klebsiella*, which were supported by path analysis (Fig. 5). *Cellulomonas* may have a direct effect on substrate degradation, FPase activity, and CMCase. Notably, most *Cellulomonas* species, as keystone organisms, encode β-glucosidase and endoglucanase genes, suggesting that cellulose hydrolysis reactions in *Cellulomonas* play an integral role in cellulose degradation within the LD community. Other studies also found that *Cellulomonas* was important for cellulose hydrolysis (50, 51). These results further demonstrate that dilution-to-extinction reduced microbial community diversity and altered the interaction of cellulolytic microbial communities, thereby affecting the cellulose degradation.

#### *Cellulomonas* was the key species for cellulose degradation in the LD community

To further confirm the effectiveness of the synthetic microbial community and elucidate the key role of *Cellulomonas* in the low-diversity (LD) consortium, we successfully isolated a *Cellulomonas* strain from the LD community. Its 16S rRNA gene sequence is provided in Text S2. We established a cellulose degradation system using this pure culture and evaluated its degradation efficiency along with the activities of relevant cellulolytic enzymes. In the 10-day short-term degradation experiment (Fig. S3), the substrate degradation rate by the *Cellulomonas* strain was almost zero during the first 4 days, and the key cellulase activities (FPase and BGL) were also almost undetectable. A sharp increase in degradation was observed between days 4 and 5, reaching 25.96%, and finally achieving 35.41% by day 10—slightly lower than the 36.42% degradation observed in the LD community (Fig. 3a). In contrast, the LD community exhibited rapid substrate degradation predominantly within the first 2 days, with cellulolytic enzyme activities already reaching high levels by day 2 (Fig. 3a). These results indicate that although the final degradation efficiency of the *Cellulomonas* monoculture is comparable to that of the LD community, its initiation and progression of cellulose degradation are markedly delayed when confronted with complex cellulosic substrates. These findings further confirmed the efficiency advantage of the LD community in cellulose degradation. We speculate that this advantage may be due to the synergistic metabolic interactions among microorganisms within the community. During the early stage of degradation, *Cellulomonas* likely benefits from cross-feeding, acquiring amino acids or other essential nutrients from other members, thereby accelerating the overall cellulose degradation process (47).

### Conclusion

The HD and LD effective lignocellulose-degrading communities have been successfully constructed by dilution-to-stimulation and dilution-to-extinction. The LD community exhibited higher substrate degradation rates, CMCase, and FPase activities than the HD community. The composition of the microbial community was significantly different in HD and LD communities. Reducing diversity shifted the key microbes and the microbial co-occurrence network during cellulose degradation. *Sphingobacterium*, *Pseudoxanthomonas*, and *Devosia* were key players in the HD community. *Cellulomonas* played a significant role in the LD community. Reducing community diversity strengthens the cooperation among functional microbes. This study provides a reference for the design of cellulolytic microbial synthetic communities and also helps to promote the efficient and high-value application of cellulosic biomass in the future.

## Data Availability

The metagenomic and metatranscriptomics sequencing data from this study have been deposited to NCBI under the BioProject accession numbers PRJNA1405287 and PRJNA1404889.

## SUPPLEMENTAL MATERIAL

Supplemental material aem.02376-25-s0001.docx Fig. S1 to S3; Text S1 and S2.

Supplemental tables aem.02376-25-s0002.xlsx Tables S1 and S2.

ASM does not own the copyrights to Supplemental Material that may be linked to, or accessed through, an article. The authors have granted ASM a non-exclusive, world-wide license to publish the Supplemental Material files. Please contact the corresponding author directly for reuse.
