# Supplementary material for: Microecological insight into the microbial structure, key cellulolytic community, and microbial interaction during cellulose degradation in high-diversity and low-diversity communities
Source: Appl Environ Microbiol. 2026 Feb 18;92(3):e02376-25. doi: 10.1128/aem.02376-25 (PMC12997860; doi:10.1128/aem.02376-25)
Supplement: Supplemental material — Fig. S1 to S3; Text S1 and S2. [file aem.02376-25-s0001.docx]

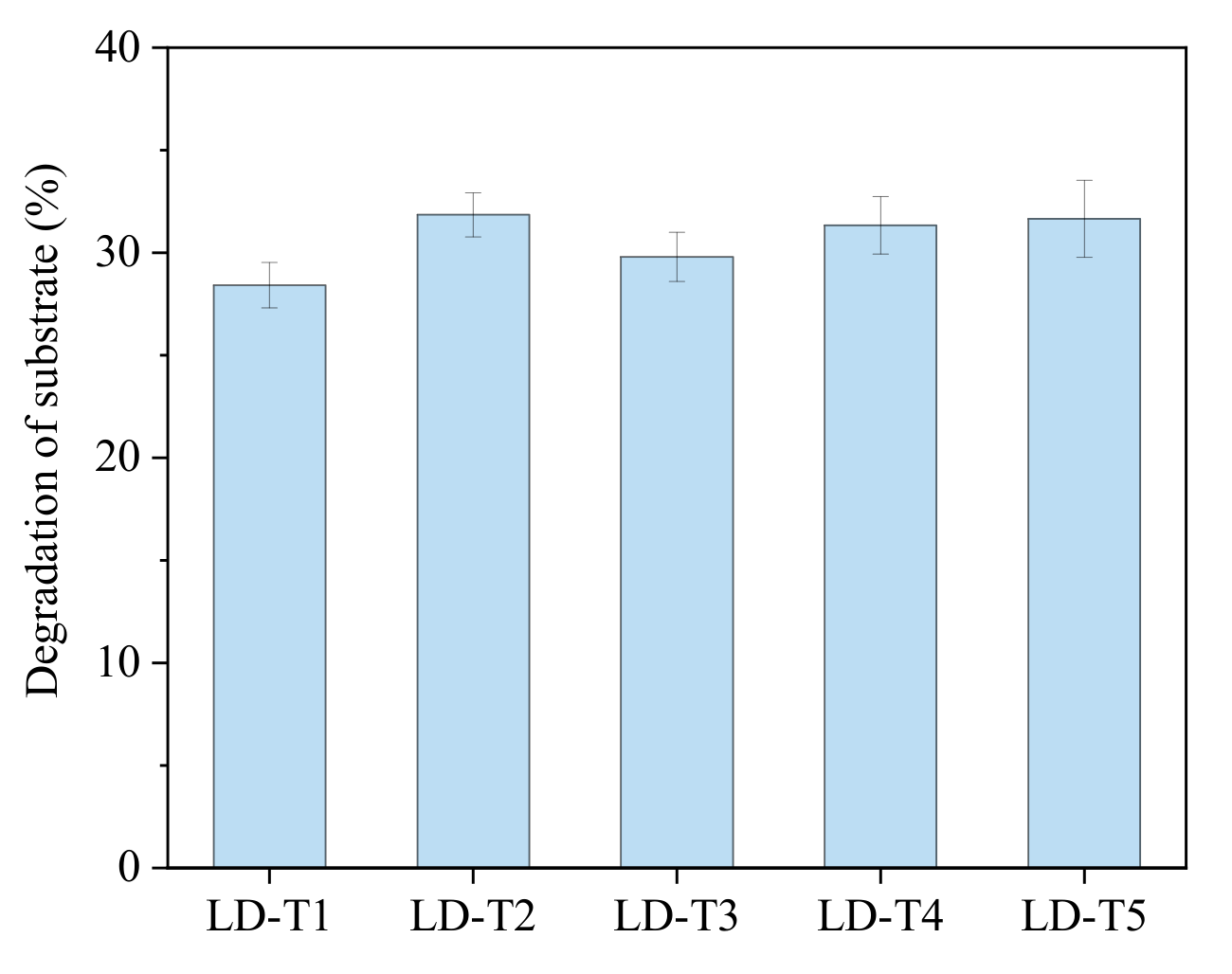


Fig. S1 The degradation of substrate along the transfers of the low-diversity community.


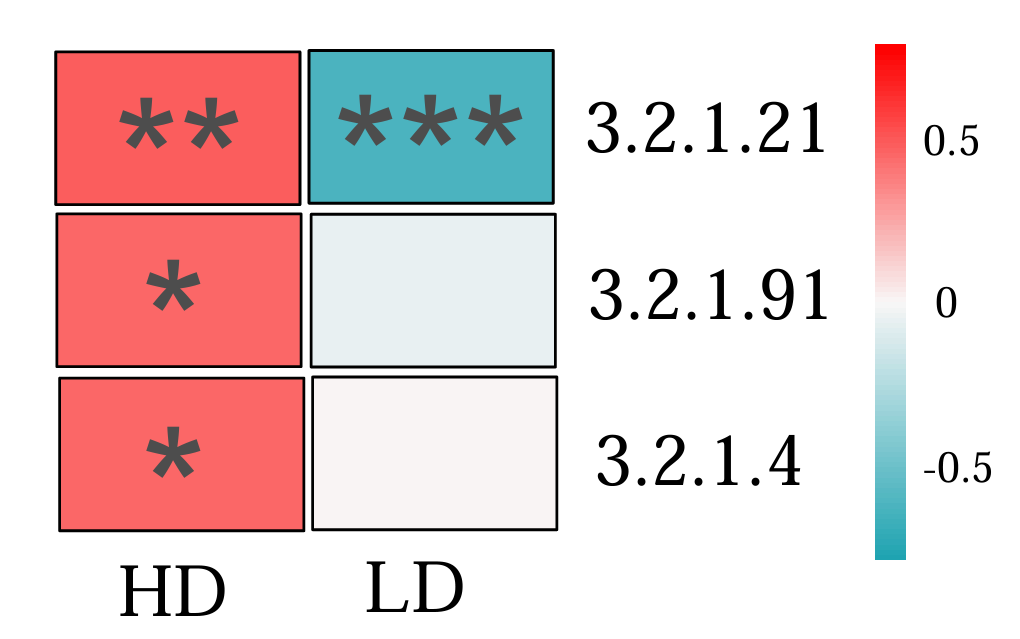


Fig. S2 Correlation analysis of the gene level and transcriptional level of cellulose hydrolysis-related enzymes


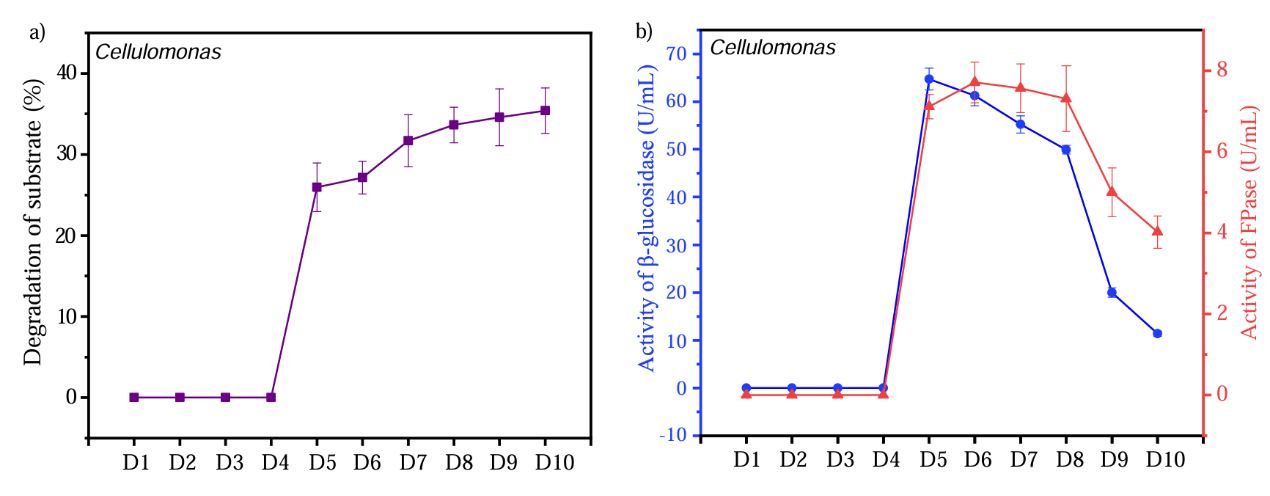


Fig. S3 **Degradation of substrate, activity of key cellulolytic enzyme during lignocellulose substrate degradation of *Cellulomonas*.** (a) Degradation of substrate. (b) Activity of key cellulolytic enzyme (FPase and β-glucosidase).

**Text S1** **DNA and RNA extraction quality control and metagenomic and metatranscriptional analysis steps**

**1. Metagenomic analysis**

**1.1 DNA extraction**

Total DNA was extracted using the E.Z.N.A.® stool DNA Kit (Omega Bio-tek, Norcross, GA, U.S.) according to manufacturer’s protocols. Concentration and purity of extracted DNA was determined with SynergyHTX and NanoDrop2000, respectively. DNA quality was checked on 1% agarose gel.

**1.2 Metagenomic sequencing**

DNA extract was fragmented to an average size of about 350 bp using Covaris M220 (Gene Company Limited, China) for paired-end library construction. Paired-end library was constructed using NEXTFLEX
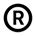
 Rapid DNA-Seq (Bioo Scientific, Austin, TX, USA). Paired-end sequencing was performed on Illumina NovaSeq™ X Plus (Illumina Inc., San Diego, CA, USA) at Majorbio Bio-Pharm Technology Co., Ltd. (Shanghai, China) using NovaSeq X Series 25B Reagent Kit according to the manufacturer’s instructions ([www.illumina.com](http://www.illumina.com)).

**1.3 Processing of metagenome sequencing data**

The data were analyzed on the free online platform of Majorbio Cloud Platform (https://cloud.majorbio.com/). Briefly, the raw sequencing reads were trimmed of adapters, and low-quality reads (length<50 bp or with average quality value <20) were removed by fastp (https://github.com/OpenGene/fastp). The quality-filtered data were assembled using MEGAHIT (https://github.com/voutcn/megahit). Contigs with a length ≥ 300 bp were selected as the final assembling result. Open reading frames (ORFs) from each assembled contigs were predicted using Prodigal（https://github.com/hyattpd/Prodigal）and a length ≥ 100 bp ORFs were retrieved. A non-redundant gene catalog was constructed using CD-HIT (http://weizhongli-lab.org/cd-hit/) with 90% sequence identity and 90% coverage. Gene abundance for a certain sample was eatimated by SOAPaligner (https://github.com/ShujiaHuang/SOAPaligner) with 95% identity.

**2. Metatranscriptional analysis**

**2.1 RNA extraction**

Total RNA was extracted using TRIzol®Reagent according the manufacturer’s instructions. Then RNA quality was determined by 5300 Bioanalyser (Agilent) and quantified using the ND-2000 (NanoDrop Technologies). Only high-quality RNA sample(OD260/280=1.8~2.2, OD260/230≥2.0, RQN≥6.5, 28S:18S≥1.0,>1μg) was used to construct sequencing library.

**2.2 Library preparation and Sequencing**

RNA purification, reverse transcription, library construction and sequencing were performed at Shanghai Majorbio Bio-pharm Biotechnology Co.,Ltd.(Shanghai,China) according to the manufacturer’s instructions. The RNA-seq transcriptome librariy was prepared following Illumina®Stranded Mrna Prep,Ligation (SanDiego, CA) using 1μg of total RNA. Shortly, messenger RNA was isolated according to polyA selection method by oligo(dT) beads and then fragmented by fragmentation buffer firstly. Secondly double-stranded cDNA was synthesized using a SuperScript double-stranded cDNA synthesis kit (Invitrogen, CA) with random hexamer primers. Then the synthesized cDNA was subjected to end-repair, phosphorylation and adapter addition according to library construction protocol. Libraries were size selected for cDNA target fragments of 300bp on 2% Low Range Ultra Agarose followed by PCR amplified using Phusion DNA polymerase (NEB) for 15 PCR cycles. After quantified by Qubit 4.0, the sequencing library was performed on NovaSeq X Plus platform (PE150) using NovaSeq Reagent Kit.

**2.3 Quality control and Read mapping**

The raw paired end reads were trimmed and quality controlled by fastp with default parameters. Then clean reads were separately aligned to reference genome with orientation mode using HISAT2 software. The mapped reads of each sample were assembled by StringTie in a reference-based approach.

**3. Functional annotation**

Searching against the KEGG (Kyoto Encyclopedia of Genes and Genomes) database was conducted for functional annotation. The coding genes for denitrification-related enzymes were identified according to KEGG PATHWAY database and the abundance was evaluated by the matched reads (reads count and relative reads number).

**Text S2 The *16S* rRNA gene sequence of isolate** ***Cellulomonas***

TGCAAGTCGAACGGTGAAGGTCAGCTTGCTGACTGGATCAGTGGCGAACGGGTGAGTAACACGTGAGCAACCTACCCTTCACTCTGGGATAAGCCTTGGAAACGGGGTCTAATACCGGATACGAGACGCACGGGCATCTGTAGCGTCTGGAAAGATTTATCGGTGGGGGATGGGCTCGCGGCCTATCAGCTTGTTGGTGGGGTAATGGCCTACCAAGGCGACGACGGGTAGCCGGCCTGAGAGGGCGACCGGCCACACTGGGACTGAGACACGGCCCAGACTCCTACGGGAGGCAGCAGTGGGGAATATTGCACAATGGGCGAAAGCCTGATGCAGCGACGCCGCGTGCGGGATGACGGCCTTCGGGTTGTAAACCGCTTTCAGCAGGGAAGAAGCGCAAGTGACGGTACCTGCAGAAGAAGCGCCGGCTAACTACGTGCCAGCAGCCGCGGTAATACGTAGGGCGCAAGCGTTGTCCGGAATTATTGGGCGTAAAGAGCTCGTAGGCGGTTTGTCGCGTCTGCTGTGAAAACCTCAGGCTCAACCTGGGGCTTGCAGTGGGTACGGGCAGACTAGAGTGCGGTAGGGGTGACTGGAATTCCTGGTGTAGCGGTGGAATGCGCAGATATCAGGAGGAACACCGATGGCGAAGGCAGGTCACTGGGCCGCAACTGACGCTGAGGAGCGAAAGCATGGGGAGCGAACAGGATTAGATACCCTGGTAGTCCATGCCGTAAACGTTGGGCACTAGGTGTGGGGTCCATTCCACGGATTCCGTGCCGCAGCAAACGCATTAAGTGCCCCGCCTGGGGAGTACGGCCGCAAGGCTAAAACTCAAAGAAATTGACGGGGGCCCGCACAAGCGGCGGAGCATGCGGATTAATTCGATGCAACGCGAAGAACCTTACCAAGGCTTGACATACACCGGAAACGTGCAGAGATGTGCGCCCCGCAAGGTCGGTGTACAGGTGGTGCATGGTTGTCGTCAGCTCGTGTCGTGAGATGTTGGGTTAAGTCCCGCAACGAGCGCAACCCTCGTCCCATGTTGCCAGCGGGTTATGCCGGGGACTCATGGGAGACTGCCGGGGTCAACTCGGAGGAAGGTGGGGATGACGTCAAATCATCATGCCCCTTATGTCTTGGGCTTCACGCATGCTACAATGGCCGGTACAAAGGGCTGCGATACCGCGAGGTGGAGCGAATCCCAAAAAGCCGGTCTCAGTTCGGATTGGGGTCTGCAACTCGACCCCATGAAGTCGGAGTCGCTAGTAATCGCAGATCAGCAACGCTGCGGTGAATACGTTCCCGGGCCTTGTACACACCGCCCGTCAAGTCATGAAAGTCGGTAACACCCGAAGCCGATGGCCCAACCGTAAGGGGGGAGTCGTCGAA
